# The Effect of Age on Technique Variability and Outcome Variability during a Leg Press

**DOI:** 10.1371/journal.pone.0163764

**Published:** 2016-10-04

**Authors:** Cassie Wilson, Oliver J. Perkin, Miranda P. McGuigan, Keith A. Stokes

**Affiliations:** 1 Department for Health, University of Bath, Bath, United Kingdom; 2 Arthritis Research UK Centre for Sport, Exercise and Osteoarthritis, Bath, United Kingdom; Duke University, UNITED STATES

## Abstract

The aim of this study was to determine the effect of aging on power generation and joint coordination during a leg press, in order to increase understanding of how functional movements are affected during the aging process. 44 older and 24 younger adults performed eight sub-maximal power repetitions on a seated leg press dynamometer. Peak power and velocity (at 40% maximum resistance) were measured along with the coordination (coupling angle) of the lower limb joints using the vector coding technique. The younger adults produced significantly greater peak power than the older adults (mean ± SD; 762 W ± 245 vs 361 W ± 162, p < 0.01) and at higher peak velocities (mean ± SD; 1.37 m/s ± 0.05 vs 1.00 m/s ± 0.06, p < 0.01). The older adults produced less consistent values of peak power than younger adults, evidenced by a higher coefficient of variation (mean ± SD; 7.6% ± 5.2 vs 5.0% ± 3.0, p < 0.01), however, there was significantly less variability in the coupling angles displayed by the older adults compared to the younger adults (mean ± SD; 2.0° ± 1.1 vs 3.5° ± 2.7, p < 0.01 (ankle-knee); 1.7° ± 0.6 vs 4.1° ± 3.0, p < 0.01 (knee-hip)). The results of this study demonstrate that older adults display higher outcome variability but lower variability in technique (coordination). The more rigid movement strategies displayed by the older adults potentially reflects an increased risk of overuse injury due to repetitive demands on the same structures, or the reduced ability to respond to unexpected situations due to a lack of flexibility in joint control.

## Introduction

Deterioration of motor performance occurs during the process of healthy aging, and the loss of physical function may be better predicted by an individual’s muscle power (i.e., the product of the force and velocity of muscle contraction) rather than muscle strength (i.e., the maximal force that a muscle can generate) [1]. In multi-joint movements, muscle coordination determines the effectiveness of the whole limb mechanics, with poor coordination leading to inefficiencies and loss of power. In such movements the motion of one joint (e.g. knee) influences the motion of an adjacent joint, and therefore the study of isolated joints does not effectively capture the complexity of the coordinated motion of components of the body [2]. Investigating the relationship between joints (e.g. knee and hip) could therefore provide valuable insights into the essential timing and sequencing of movement control [3].

Movement variability during repetitions of the same task is often considered as an unwanted source of error that should be eliminated or reduced [4,5]. However, movement variability also corresponds to subtle functional changes in the way movements are performed, which allow the human body flexibility and adaptability to cope with variable external conditions [6]. Conventional methods of quantifying movement variability (such as standard deviation or intra-class correlation) can only quantify overall variability in parameters of a single joint and may not allow the identification of subtle changes in movement patterns. More recent approaches which analyse multi-joint coordinative features provide alternative ways to explore the nature of movement variability and provide information on the flexibility of the body to control multiple joints.

When considering functional movement tasks, care should be taken not to confuse variability in task outcome with variability in how the task is carried out. In this context, low variability in the outcome of a task does not necessarily indicate a low variability in movement strategy or technique (such as joint coordination measures). The relationship between joint coordination variability and task outcome has been previously investigated, and in well learned movements low levels of outcome variability are associated with high joint coordination variability [7,8]. This finding suggests that the variability in coordination patterns may provide flexibility such that the body can search for the optimal solution to carry out a task whereas variability in outcome measure likely represents a decline in the performance of a task.

In terms of maintaining overall stability, elderly individuals have been shown to adopt more rigid (consistent) coordination strategies compared with younger individuals when stability is challenged [9]. Hsu et al. [10] identified that healthy aging adults lose the compensatory strategy of flexibly controlling multiple joints when trying to gain stability after recovering from a balance perturbation. This behaviour was attributed to an inability to control the multiple degrees of freedom present during performance of a whole-body coordination task, due to its greater requirements of central processing and afferent update from the periphery. During a rapid upper body reaching task, Sleimen-Malkoun et al. [11] similarly observed results that suggested a lack of adaptability and therefore variability in the behavioural responses with aging.

Much of the literature on coordination and its associated variability of the lower limbs in older adults has focused on balance recovery with only limited research on planned dynamic movements. Since planned dynamic movements feature in Activities of Daily Living (ADL) undertaken by older adults, the ability of muscles to proactively generate power is of great consequence [1]. In a study of vertical jumping, Haguenauer et al. [12] found that the sequential pattern of joint coordination commonly observed in younger adults was not demonstrated in older adults. Haguenauer et al. [12] hypothesised that these differences in joint coordination patterns between age groups were due to an augmented rigidity of the multi-joint system, however this was not directly quantified. Whilst jumping is not a common exercise used in an older population, pneumatic leg press machines are commonly adopted in both rehabilitation and resistance training settings. A leg press is essentially the same movement of the lower limbs as a vertical jump, just in a more controlled way, without the addition of arm swing impacting on variability, and therefore leg press machines provide a useful tool for assessing power generating capabilities and joint coordination patterns during a low impact dynamic task [13].

The aim of this study was to determine the effect of aging on the power generation and joint coordination during a leg press in order to increase understanding of how functional movements are affected during the aging process. It was hypothesised that older adults would display higher variability in outcome (power output) measure and lower variability in technique (joint coordination) measures compared to younger adults.

## Methods

### Participants

Healthy, older (65–80 years) and younger (20–35 years) adults were recruited for the study. Younger participants were students and staff recruited from the University population, and older participants were recruited from the surrounding community. Ethical approval for the study was obtained from the University of Bath’s Research Ethics Approval Committee for Health (REACH) and each participant gave written informed consent prior to the onset of the data collections. Prior to the main trials, participants had their height and mass measured. To assess lower extremity function the Short Physical Performance Battery (SBBT) (timed 2.4-m walk, 5 timed repetitive chair stands and tests of standing balance) was carried out. This battery of tests has previously been used in community settings [14] and is scored on a scale of 0 to 12. Only individuals scoring 8 or above and not scoring zero on any particular test were included in the study. The group mean score ± SD for the battery of tests was 11 ± 1 with no participants failing (Table 1). A preliminary trial was then completed to familiarise the participants with the leg press equipment and testing protocol and to establish their one repetition maximum (1-RM) load on which the resistance for the testing protocol was based. Participant characteristics are presented in Table 1**.**

**Table 1:** Participants’ characteristics.

	Older		Younger	
	♂ (*n* = 22)	♀ (*n* = 22)	♂ (*n* = 13)	♀ (*n* = 11)
Age (years)	70 (4)	69 (3)	25 (4)	25 (3)
Body mass (kg)	75.8 (11.7)	62.1 (11.7)	73.8 (7.5)	62.0 (5.8)
BMI (kg/m^2^)	24.8 (3.4)	23.8 (3.4)	22.7 (1.6)	21.4 (1.8)
SBBT score	10(1)	11(1)	11(1)	11(1)

Data presented as mean (standard deviation). BMI = Body mass index.

### Experimental Procedure

A pneumatic Keiser A420 seated leg press dynamometer (Keiser®, Fresno, CA) was used to measure lower limb power and velocity characteristics using the manufacturer’s software. The seat position was set such that pre-repetition knee angle was approximately 90° and the participant was comfortable, with the same seat position used in both the preliminary and main trials. During the preliminary trial, which was completed seven days prior to the main trial, participants performed a 1-RM test in which discrete repetitions to failure were attempted at participant-selected increments in resistance. Repetition velocity and rest periods between repetitions were self-selected, with participants instructed to aim to reach their 1-RM within 20 repetitions. During the main trial participants completed an incremental 10 repetition power test (with the 10^th^ repetition at the previously achieved 1-RM) as a warm up. Following a 10 minute rest, participants performed 8 discrete repetitions of the leg press exercise at 40% 1-RM resistance with 60 seconds recovery between repetitions. Participants were instructed to perform the repetitions as fast as possible.

### Data Collection & Processing

Peak power and velocity at peak power of the leg press footplate were determined for each of the 8 40% 1-RM repetitions of the main trial. The calculation of these variables discarded the first and last 5% of the footplate displacement to allow for any uncertainties at the ends of the repetition to be discarded from further calculations. A Cartesian Optoelectronic Dynamic Anthropometer (CODA) 6.30B-CX1 motion analysis system located to the left of the leg press dynamometer was used to obtain three-dimensional (3D) coordinate data (sample rate: 200 Hz) during the 8 repetitions. The CODA system was aligned such that the y-axis of the global coordinate system was defined by the anterior-posterior axis of the leg press dynamometer. Active markers were secured to the lateral aspect of the left side of the body on the fifth metatarsal phalangeal joint (MTP5), the lateral malleolus, the lateral epicondyle, the greater trochanter and the acromion process. Following data collection, the 3D coordinate data of the accepted trials were reduced to two-dimensions (2D) (y: anterior-posterior and z: vertical).

### Data Analysis

In order to define the period of interest (i.e., the leg extension phase of the leg press) the MTP5 marker velocity in the y-axis was used. The period of interest started once the velocity of the MTP5 reached a threshold of 0.1 m/s and ended when the velocity dropped below 0.5 m/s. These values were chosen to ensure that only data during the leg extension phase where obtained. Sagittal plane joint angles for the left ankle (plantar-dorsiflexion), knee (flexion-extension) and hip (flexion-extension) were derived for each leg press as 2D projections on the y-z plane. The joint angle data were subsequently low pass filtered using a cut-off frequency of 8 Hz, and normalised to 100% of the leg extension phase. For some participants only 7 trials were used for subsequent analysis due to trial data being incomplete as a result of markers not being visible for the whole movement.

**Fig 1:**
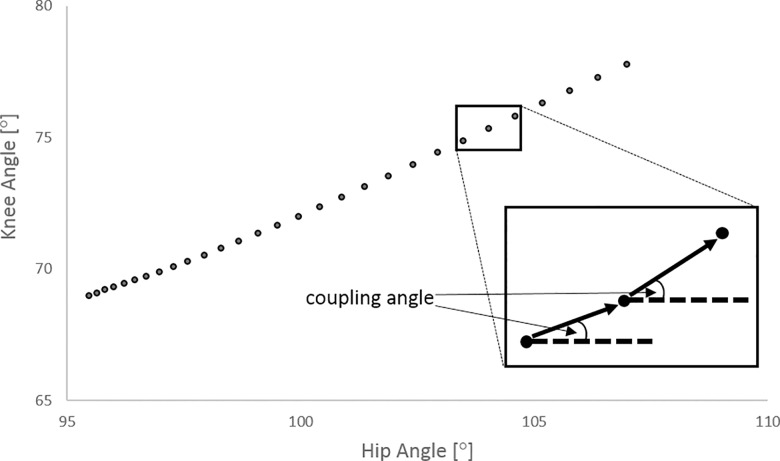
Schematic representation of Vector Coding.

Vector coding [15] is a technique used to quantify coordination between joints and can provide data in a potentially more interpretable form than other techniques [16]. This technique was therefore used to quantify coordination patterns of two pairs of joints (referred to as couplings); (i) flexion-extension of the ankle–knee and (ii) flexion-extension of the knee–hip. In order to apply the vector coding technique, angle-angle plots were created for each coupling whereby the ordinate displayed proximal joint angles and the abscissa displayed distal joint angles. Coordination was defined as the magnitude of the ‘coupling angles’ which were determined using the angle of the vector joining two adjacent points on the angle-angle plots (Fig 1). The range of values for each of the coupling angles was between 0° and 180°. The standard deviation of coupling angle across repetitions was calculated for each percent of the leg extension phase, with a mean value of the standard deviation across the leg extension phase being subsequently calculated for each participant. This provided a measure of between repetition (intra-participant) coordination variability [17]. A mean value of the intra-participant coordination variability during the first 50% and last 50% the leg extension phase was also calculated in order to look at differences within different phases of the movement. Because these data are directional and thus classified as a circular variable, the arithmetic mean and standard deviation does not provide an appropriate descriptive measure for these data. Therefore, the intra-individual trial means and standard deviations were calculated using circular statistics [18].

### Statistical analysis

Whilst sex effects were not the focus of the study, it was important to consider them in order to be able to interpret fully the overall findings, and thus a one-way analysis of variance (ANOVA) was employed (age main effect; sex main effect; age–sex interaction effect) to investigate any differences in; (i) peak power (at 40% maximum resistance), (ii) velocity at peak power, (iii) intra-individual variability of peak power (assessed using the coefficient of variation), (iv) intra-individual variability of velocity at peak power (assessed using the coefficient of variation) and (v) coupling angles. Where significant interaction effects were identified, independent t-tests were employed post-hoc to examine where the significant differences existed. Statistically significant differences were accepted at p < 0.05. Effect size was calculated according to Cohen [19], with d>0.8 considered a large effect.

## Results

**Fig 2:**
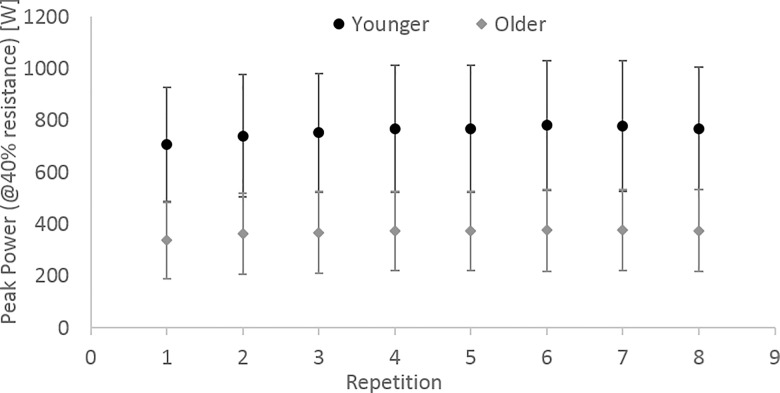
Peak power (at 40% resistance) across repetitions for younger and older adults.

The younger adults produced significantly greater peak power (at 40% 1-RM) than the older adults (mean ± SD; 762 W ± 245 vs 361 W ± 162, p < 0.01) and at higher peak velocities (mean ± SD; 1.37 m/s ± 0.05 vs 1.00 m/s ± 0.06, p < 0.01) (Table 2). In addition, males produced higher peak power than females (mean ± SD; 652 W ± 267 vs 355 W ± 164, p < 0.01) (Table 2). There were significant interaction effects and post hoc t-tests revealed significant differences in peak power produced between; younger males and older males (p < 0.01), younger females and older females (p < 0.01), younger males and younger females (p < 0.01) and older males and older females (p < 0.01). Peak power did not decline over the 8 repetitions (Fig 2).

**Table 2:** Peak Power and velocity (at 40% resistance) during the leg extension phase.

	Older		Younger	
	♂ (*n* = 22)	♀ (*n* = 22)	♂ (*n* = 13)	♀ (*n* = 11)
Peak Power (W)	471 (152)*^	267 (95)*	959 (104)^	530 (127)
Peak Velocity (m/s)	1.16 (0.23)*	0.89 (0.21)*	1.43 (0.07)	1.30 (0.20)

Data presented as mean (standard deviation).

* denotes difference between age groups (p < 0.01),

^ denotes difference between sex (p < 0.01).

The intra-individual variability of peak power, assessed using the coefficient of variation across the set of repetitions for each participant, demonstrated that younger adults were able to produce more consistent peak power values than older adults; the younger adults demonstrated a mean coefficient of variation ± SD of 5.0% ± 3.0 compared to the older adults who displayed a mean coefficient of variation ± SD of 7.6% ± 5.2 (p < 0.01). There was no significant main effect for sex (p = 0.94) and no significant interaction (p = 0.42).

**Fig 3:**
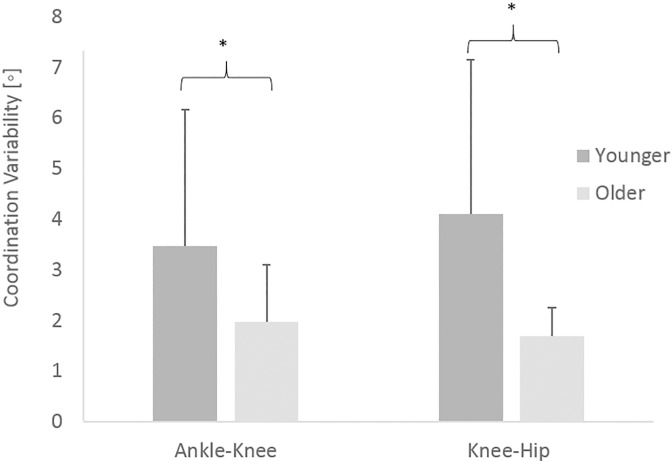
Coordination variability for the ankle-knee and knee-hip couplings for the younger and older adults. * denotes significant difference between age groups (p < 0.01).

**Fig 4:**
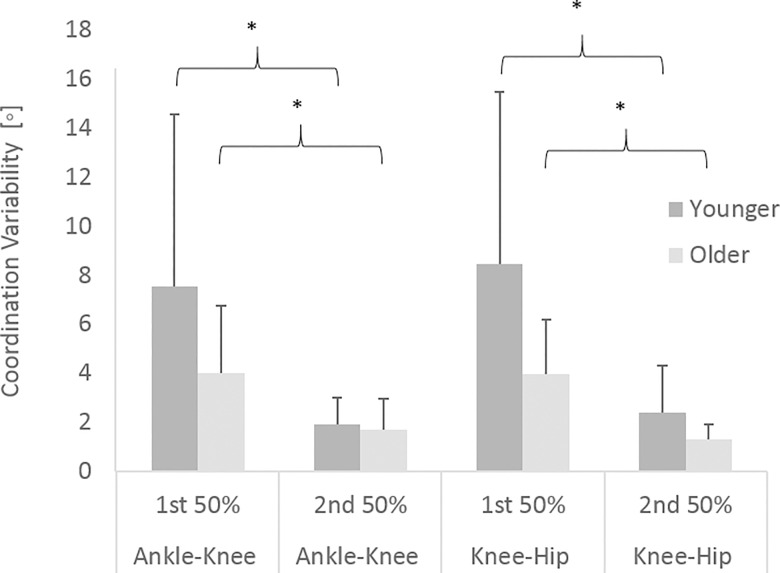
Coordination variability for the ankle-knee and knee-hip couplings for the first and last 50% of the leg press movement. * denotes significant difference between phases of the movement (p < 0.01).

**Fig 5:**
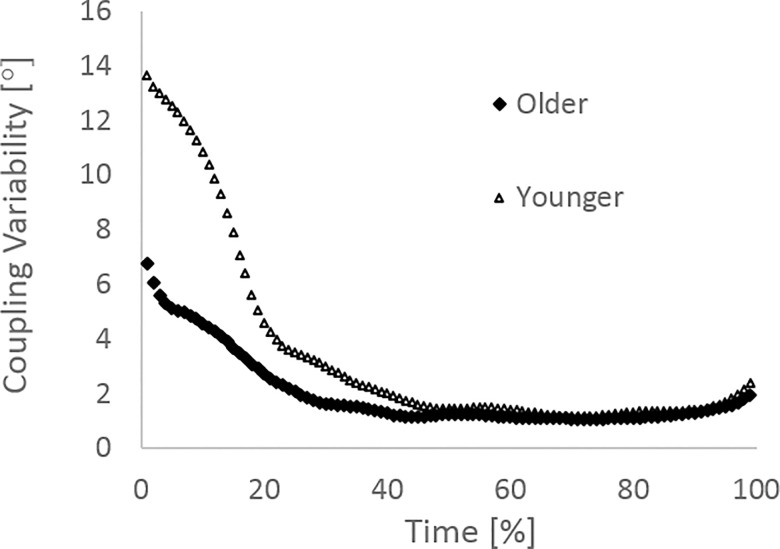
Coordination variability as a % of leg extension phase for the ankle-knee coupling.

**Fig 6:**
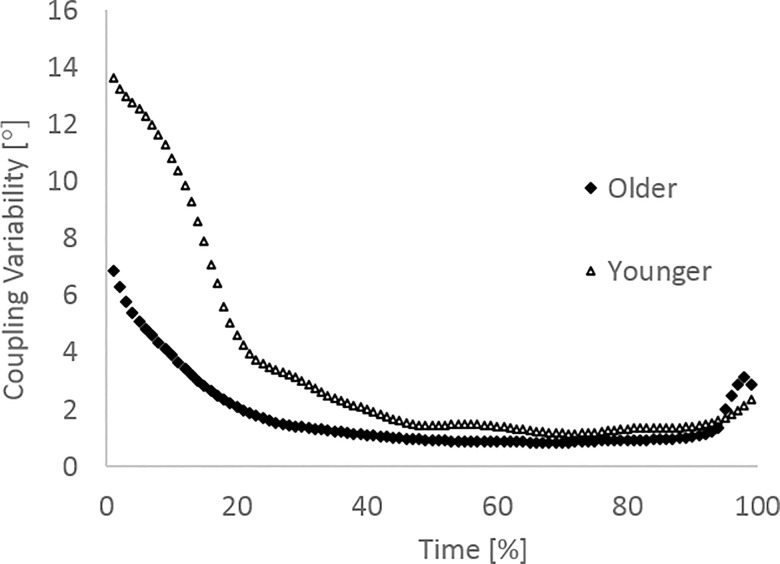
Coordination variability as a % of leg extension phase for the knee-hip coupling.

In terms of the coordination strategies adopted by the participants, Fig 3 shows there was significantly more intra-participant variability displayed by the younger adults than the older adults for both the ankle-knee (mean ± SD; 3.5° ± 2.7 vs 2.0° ± 1.1, p < 0.01) and knee-hip couplings (mean ± SD; 4.1° ± 3.0 vs 1.7° ± 0.6, p < 0.01). Effect sizes of 0.8 and 1.1 for the ankle-knee and knee-hip couplings respectively demonstrate a large effect. There was a significant main effect for sex for both couplings with males displaying higher levels of intra-participant variability compared to females for both the ankle-knee coupling (mean ± SD; 2.9° ± 2.4 vs 2.05° ± 1.2, p = 0.03) and the knee-hip coupling (mean ± SD; 2.9° ± 2.6 vs 2.1° ± 1.6, p = 0.04), however there was no significant interaction effect for either coupling (p = 0.08 (ankle-knee); p = 0.12 (knee-hip)). When broken down into the first and last 50% of the leg press movement the results showed significantly higher coordination variability in the first half of the movement than the second half for both younger adults and older adults (Fig 4). Figs 5 and 6 show the mean coordination variability throughout the leg extension phase for the younger and older adults for the ankle-knee and knee-hip couplings respectively.

## Discussion

The major finding of this investigation was that older adults display higher levels of outcome variability (i.e., variability in what has been achieved) and lower levels of technique variability (i.e., variability in the joint coordination strategies used to achieve the movement) than younger adults during leg press performance. These findings support the hypotheses of the study and suggest that older adults are less consistent in task outcome and adopt more rigid coordination patterns during a dynamic task.

It was hypothesised that the older adults would be more variable in their ability to produce power because of age-related changes in neuromuscular function, and the results support this hypothesis. Neuromuscular activation changes with age [20] and this change has previously been linked to increased variability in motor output, which impairs the functional capabilities of older adults compared with younger adults [21]. Specifically, greater variability in the motor unit discharge seen in older adults is associated with greater force variability [22]. The higher variability seen in older adults can be decreased by training [21,22] and this supports a further explanation for increased variability in outcome being a result of the experience / training level of older adults in performing such exercises. There has been a wealth of literature on expert and novice performers and many studies have identified that expert performers are capable of producing more consistent outcomes (e.g. Pojman et al. [23]). Whilst the younger adults in this study cannot be classified as experts and similarly the older adults as novices in the specific leg press task, a difference in level of general training between older and younger adults in related activities was identified and may account for some of the differences found. The study’s protocol deliberately did not include an extensive familiarisation with the leg press procedure in order that differences in coordination during a relatively novel task were identified. The results of the study suggest that sex does not influence intra-participant variability age differences in peak power output as no differences in outcome variability between male and female participants were identified.

In terms of the lower variability in coordination strategies, the majority of the previous research has focused on balance recovery. Within this body of literature it has been highlighted that when the human body is challenged (from a balance perspective) older adults adopt more rigid strategies to cope with the challenge [9,10]. The results of this study show a similar pattern of reduced variability in the coordination strategies adopted, most likely due to the physical demands of an explosive movement. During a fast movement at relatively low forces the lower limbs are challenged in a way which differentiates between older and younger adults. This lower joint coordination variability may be indicative of older adults being less able to control multiple joints when the body is physically challenged during such a movement [17]. The use of co-activation of the agonist and antagonist muscles [24] is likely to contribute to this more rigid coordination strategy identified in the current study however this was not assessed. The lower levels of coordination variability also potentially reflects an increased risk of overuse injury due to repetitive demands on the same structures of the human body [25]. Whilst the same interpretation regarding overuse injuries could be made for younger adults displaying similar levels of coordination variability during a repetitive movement, older adults may be more susceptible due to the higher prevalence of osteoarthritis in this age group [26]. Whilst no participants reported pain, it is possible that older adults were restricted in the use of certain coordination patterns due to their perceived risk of onset of joint pain. The higher levels of coordination variability in the first half of the leg press movement can be explained by the challenges of initiating movement of the plate, demonstrating a need for additional flexibility / adaptability in the system (human body) when physical demand is increased. Whilst sex differences were identified, with males displaying higher levels of intra-participant joint coordination variability in agreement with O’Connor and Bottum [27], no interaction effects were identified, demonstrating that the difference between the older and younger group was not influenced by sex. The findings of this study support the fact that outcome and technique variability should not be treated the same, as low variability in the outcome measure does not always indicate a low variability in technique parameters describing the movement.

Since most everyday tasks require dynamic movement, the ability of muscles to generate and sustain power is of great consequence [28]. In addition, a decline in lower limb muscle power with age has important implications for independent physical function [20]. In this study, older adults produced significantly lower peak power during the leg press. This difference in power producing capabilities between younger and older age adults is well documented in the literature [1,29] and can be attributed to multiple factors including a decline in skeletal muscle mass and a change in properties of muscle fibres [30]. Whilst sex differences were not the focus of the paper it was important to consider their effect on the overall findings. Differences in peak power between males and females were identified as expected [31], however, differences between both younger females and older females and younger males and older males were also identified and although these differences were not the same (as highlighted by a significant interaction), the influence of age remains similar for both sexes. Since peak power did not decline over the 8 repetitions, fatigue was not considered a confounding factor. This was not surprising as the repetitions were performed at 40% 1-RM resistance with one minute rest between repetitions, which represents a relatively low intensity task for any individual to perform.

In terms of the way tasks or movements are achieved by the neuromuscular system, older adults tend to adopt more cautious movement strategies that are defined by a reduction in speed of motor performance [9]. The lower velocity exhibited by the older adults in this study is consistent with previous studies which have shown older adults exhibit slower velocities in walking [32,33] and stepping-up exercises [34]. These reduced velocities may be used in an effort to facilitate stability or simply be an indicator of reduced muscle capacities [34]. In general, older adults are slower at initiating and performing movements [35].

## Conclusion

In conclusion, the results of this study demonstrate that during a relatively challenging coordination task such as a leg press, older adults display higher outcome variability but reduced variability in joint coordination strategies. The increased variability in peak power (at 40% 1-RM) can be attributed to age-related changes in neuromuscular function. The more rigid (consistent) movement strategies displayed by the older adults are likely adopted because of an inability to control the multiple degrees of freedom present during the performance of a challenging coordination task. Lower levels of coordination variability in older adults potentially reflects an increased risk of overuse injury due to repetitive demands on the same structures, or the reduced ability to respond to unexpected situations due to a lack of flexibility in joint control.
